# Influencing factors of destination choice of ski tourism enthusiasts: A means-end chain analytical perspective

**DOI:** 10.3389/fpsyg.2022.1017961

**Published:** 2022-12-09

**Authors:** Hui Sun, Xia Xie, Jun Gao, Lei Zhang

**Affiliations:** ^1^Key Laboratory of the Sustainable Development of Xinjiang's Historical and Cultural Tourism, Xinjiang University, Urumqi, China; ^2^College of Tourism, Xinjiang University, Urumqi, China; ^3^School of Tourism Management, Sun Yat-sen University, Zhuhai, China

**Keywords:** ski tourism enthusiasts, ski tourism destination, tourism destination choice, means-end chain, influencing factors

## Abstract

Ski enthusiasts are a unique group of travelers whom many travel businesses and destinations market to. Drawing on a means-end chain analytical perspective, this study explores the main factors affecting the destination choice of ski tourism enthusiasts. Three levels of main factors, including destination attribute, experience consequence, and personal value, were first explored via a soft laddering interview (*N* = 28), followed by a questionnaire survey (*N* = 339). The results show that ski resort conditions (e.g., quality of ski tracks and snow quality) are the most important attribute of the destination for ski enthusiasts, with the strongest influence on their experience sequence. In addition to the value needs of positive emotion and self-adjustment, ski tourism enthusiasts also show a strong need for self-development. This result has important guiding significance for the formulation of ski tourism destination marketing strategy.

## Introduction

The successful bid for the 24th Winter Olympic Games has cast a domestic spotlight on ice and snow sports in China. Currently, many provinces and cities in China are vigorously building ice and snow tourism destinations with high-standard ski resorts and ski tourism towns and as the main products. The public promotion and popularization of skiing are accelerating with China’s skiing market gradually moving away from a high-end sport to a mainstream popular sport. However, there remains a significant gap in the penetration rate of China’s skiing population compared with European and American developed countries (Li, 2018).

As a niche sport, ski tourism tends to exhibit sports’ spirit of freedom, teamwork, and enterprise, with strong bonds formed, resulting in a unique subculture group of ‘ski tourism enthusiasts’. This group tends to have more recognition and involvement in skiing than mass tourists. They usually invest higher costs and energy in skiing activities. Also, their tourism consumption behavior is different from mass tourists (An et al., 2019). Compared with mass tourists, ski enthusiasts have higher professional knowledge and skiing skills with a higher revisiting rate. As the pioneering tourists of ski tourism, ski enthusiasts have played an important role in discovering tourism resources, disseminating tourism information, planning tourism products, and promoting destination brand building (Li, 2018). As skiing ‘experts’, this group’s destination choice will also influence the mass tourists’ choice and image perception of specific ski tourism destinations.

Existing research on sports tourism enthusiasts has focused on sports event tourism enthusiasts. They believe that the development of sports event tourism is more significantly affected by the ‘fan economy’. On the other hand, the research vision of active sports tourism participants, such as ski tourism, is mainly concentrated on mass tourists to explore the commonality of their tourism behavior (Cao and Jiang, 2020), motivation (Christopher and Mark, 2011) and satisfaction (Lu et al., 2018). To this end, extant literature has not paid enough attention to ski tourism enthusiasts. As a niche market, ski tourism enthusiasts may show different patterns in destination selection and tourism participation behavior when compared with mass tourists (Zhang et al., 2019; Wang et al., 2020).

By adopting a mixed-method research approach, this study explored the factors influencing ski tourism enthusiasts’ destination choice from a means-end chain analytical perspective. Specifically, the specific factors at ski destination attribute level, consequence of ski consumption level, and associated personal value level were first explored *via* soft laddering interview. Then a visitor survey was conducted to examine the relationship among the three levels of factors. By doing so, this study provides empirical evidence that ski enthusiasts do have distinctive preferences for ski destinations, thus advancing extant knowledge of ski tourists. It also provides reference information for ski tourism destination operators to formulate targeted reception service plans and carry out effective marketing to attract ski enthusiasts.

## Literature review

### Means-end chain theory

The means-end chain theory was put forward by Jonathan Gutman in 1982 to understand consumers’ feelings about a specific product and service. Consumers will mentally attach different impressions to different products or services. These mental impressions form the basis for consumers’ intention to purchase and are connected to individuals’ values. Means are the tangible activities or things people are engaged in. Ends refer to the intangible value states such as happiness, security, and achievement. The model of the means-end chain explores how consumers promote the final state they want to achieve through the choice of products or services (Gutman, 1982). In the means-end chain theory, the meaning of products or services to consumers is usually segregated into three levels: product attribute, experience consequence and personal value. As seen from the theoretical framework, personal value is highly intangible, a key factor in guiding consumers’ choices and behavior (Overby et al., 2004).

Product attributes can be distinguished from different perspectives. It is more common to divide product attributes into abstract and physical attributes (Belk, 1988). Abstract attributes refer to the brand value, quality, and other intangible characteristics that consumers cannot perceive before purchasing products, while physical attributes refer to the external, tangible characteristics of products that consumers can see, such as product color, size, price, weight and so on. Some researchers believe that the product attributes can be divided into internal and external attributes (Reynolds and Olson, 2001). Internal attributes refer to attributes that consumers cannot perceive and experience before purchasing products, such as labor value and product quality. In contrast, external attributes refer to attributes such as product reputation and brand value that consumers can perceive before purchasing (Reynolds and Olson, 2001).

Research has classified the consequences of consumption into functional and psychological. The functional consequence is the most direct product experience that consumers get from products. The psychological consequence refers to the psychological satisfaction obtained by consumers from products, such as the psychological recognition of the society by buying and using brand-name products (Lai, 1995).

Personal value refers to the core belief or ultimate goal that consumers seek to achieve through purchase and consumption behavior (Westerlund, 2007). Value is an important factor affecting and even determining individual behavior, including consumption behavior (Xing et al., 2018). Personal values are able to stimulate consumers’ interest and desire for a product or service, thus affecting individual behavior.

Means-end chain theory explores why and how product attributes are important by evaluating the correlation between product attributes and personal value. It can also describe the path of the connection between the attributes of products and the final value and purpose expected by consumers.

### Destination selection by ski tourists

Presently, there is a burgeoning field of research on tourism decision-making and destination selection. Early works primarily focus on tourists’ decision-making using conceptual models such as the tourism decision-making process model (Um and Crompton, 1990), destination cognition, and choice model (Gutman, 1982; Klenosky et al., 1993). Overall, four major types of factors affect tourists’ decision-making, namely tourism stimulation, personal needs and desires, external factors, and destination characteristics (Schmoll, 1977). Unlike earlier studies that tend to provide a systematic understanding of tourists’ destination choice, more recent studies tend to focus on specific factors influencing destination choice, such as destination images of tourists’ social network members (Pan et al., 2021), risk perception (Karl, 2018; Karl et al., 2020), and social media (Tham et al., 2020).

In ski tourism destination selection, snow resort conditions (François et al., 2014), interpersonal atmosphere (Mlađenović and Jovanović, 2019), and ski terrain (He and Luo, 2020) have been proven to influence destination choice. More recent studies have explored the impact of weather and climatic factors on ski tourists’ destination choice (e.g., Witting and Schmude, 2019; Witting et al., 2021). As already mentioned, ski tourists are not homogeneous, therefore the importance of relevant factors varies between different segments. Konu et al. (2011) and Humphreys (2014) find that sports experience is the primary consideration for ski tourists with interest in skiing. Notably, Konu et al. (2011) segment Finnish ski resort visitors according to ski destination choice attributes into six groups, namely passive tourists, cross-country skiers, want-it-all, all-but-downhill skiing, sports seekers, and relaxation seekers. For different types of ski tourists, there are also differences in the deciding factors regarding their destination choice. Passive participants, such as older people in family-related tourism activities, tend to focus on the role of ski tourism in promoting interpersonal relationships (Kang et al., 2011; Otoo and Kim, 2020). For professional skiers, the impact of sports experience is significantly stronger than other factors. Unbehaun et al. (2008) surveyed 540 professional skiers from Vienna, Austria, and asked the respondents to make a trade-off between various combinations of destination attributes. The study found that skiers have the strongest preference for the destination attribute regarding snow conditions. When asked to weigh the extra cost and additional travel distance with the conditions (safety) of the destination’s snow field, most skiers are willing to bear additional costs and distances within a specific range. Likewise, Won et al. (2008) note that compared with less experienced skiers, advanced skiers are more concerned about snow condition and trail variety, and are less concerned about cost barriers. The current research on the influencing factors of ski tourism selection is mainly based on a specific perspective, such as tourism destination image or tourist value. There is a research gap in understanding the conceptual relationship between the influencing factors of different dimensions. Researchers usually think that the factors are completely different and independent, ignoring the conceptual relationship between the factors of various dimensions.

## Methodology

### Soft laddering interview

Data collection *via* interviews was conducted from August 11 to October 5, 2021. Ski tourism enthusiasts who have been skiing annually for the past 3–5 years were selected for one-on-one interviews. The interviewees were recruited *via* a snowball sampling approach. The authors first identified ski enthusiasts with their own networks, and these respondents were asked to recommend qualified respondents for further interviews. In this way, 28 ski enthusiasts were interviewed. The interviews were either conducted online or through face-to-face communication in ski resorts, and each lasted about 20–30 min. All interviews were recorded with the consent of the respondents. The main purpose was to identify the specific factors influencing their destination choice within the attribute-consequence-value framework. This is because the existing literature pays insufficient attention to ski enthusiasts and thus lacks detailed information.

In terms of the interview process, first, we asked the interviewees about their most frequently visited ski tourism destinations, followed by the attributes they attach most importance to when choosing ski tourism destinations, to obtain the elements at the destination attribute level. Next, the respondents were asked about the reasons for their choices to probe their motivation at the result and value level. The interview ends when the respondent cannot answer the questions further. NVivo11.0 was used to encode the elements in the transcribed interview. Data were classified and summarized according to the model framework of means-end chain theory.

### Questionnaire survey

Through the interviews, this study obtained several relevant elements of destination attributes, experience consequences, and personal value considered by ski tourism enthusiasts in destination selection. We encoded the elements in the interview text. The data are classified and summarized according to the model framework (attribute, consequence, and value) of the means-end chain theory. Finally, a total of 47 measurement elements were coded and summarized into three levels: destination attribute, experience consequence, and personal value. Among them, the destination attribute level includes 20 elements, such as snow quality and condition, the length, width, and slope of the ski tracks. The experience consequence level includes 15 elements, such as improving skills, saving time, high comfort level, and high-cost performance. The personal value level includes 12 elements, such as a sense of satisfaction, a sense of achievement, a sense of security, and a sense of belonging (see Table 1). According to the means-end chain theory, it is expected that factors at the destination attribute level would significantly affect factors at the experience consequence level, which, in turn, would impact factors at the personal value level.

**Table 1:** Measurement items of influencing factors for destination choice of ski tourism enthusiasts.

Destination attribute	Experience consequence	Personal value
A1 Snow quality and snow condition	C1 Improving skills	V1 a sense of satisfaction
A2 Length of ski track	C2 Saving time	V2 a sense of achievement
A3 Width of ski track	C3 High comfort level	V3 a sense of security
A4 Slope of snow track	C4 High-cost performance	V4 a sense of belonging
A5 Scale of ski resort	C5 High safety	V5 a sense of freedom
A6 Ski resort service	C6 High flexibility	V6 Happiness and pleasure
A7 Ski resort popularity	C7 High playability	V7 Self-challenge
A8 Crowding degree of ski resort	C8 Novelty	V8 Self-realization
A9 Ski infrastructure	C9 Relaxation	V9 Self-improvement
A10 Catering facilities	C10 Close to nature	V10 The joy of life
A11 Accommodation facilities	C11 Social atmosphere	V11 Spiritual comfort
A12 Tourism shopping	C12 Getting attention	V12 Promoting work and learning
A13 Leisure and entertainment facilities	C13 Worry-free	
A14 Traffic accessibility	C14 Stimulation	
A15 Destination cultural scenery	C15 Trust	
A16 Destination natural scenery		
A17 Ticket price of ski resort		
A18 Transportation cost		
A19 Catering cost		
A20 Accommodation cost		

The questionnaire consists of four parts. Before filling in the survey, the respondent should first answer the question—Do you go skiing every year in the past 3 years? If the answer is affirmative, the respondent will be allowed to fill in the formal questionnaire. The first part of the survey is to investigate destination attributes when choosing ski tourism destinations. The second part is the investigation of the experience consequences of ski tourism enthusiasts in ski tourism destinations. The third part investigates the personal value feelings that ski tourism enthusiasts can fulfill when they travel. Richter’s 5 points scale was adopted for these three parts. The fourth part includes collecting demographic data: gender, age, occupation, income and so on.

This study’s survey was distributed from November 15 to December 9, 2021. The respondents were ski enthusiasts who have participated in yearly ski tourism in the past 3 years. The questionnaire was distributed both in paper format in a ski resort and electronically. The reasons are as follows. First, it was difficult to identify ski enthusiasts at the resort because it hosted not only ski enthusiasts but also ordinary tourists. Second, considering the impact of Covid-19 on restricting ski enthusiasts’ mobilities, online distribution could help us cover skiing tourism fans as wide as possible. In fact, ski enthusiasts form clubs and online communities (e.g., Mu Xue Club) to communicate and exchange information, and thus can be easily approached *via* these channels. Specifically, 339 questionnaires were sent out by disseminating electronic questionnaires to ski tourism clubs and inviting ski tourism enthusiasts to fill in the survey. We deleted questionnaires with large missing values, and 198 questionaries were valid, with an effective rate of 86%. On the other hand, 175 questionnaires were distributed in Beijing’s Nanshan ski resort, with 159 questionnaires collected. Subsequently, we eliminated questionnaires that were not seriously filled in (e.g., same options across the questionnaire), and obtained 141 valid ones, with an effective rate of 88%. This led of a total valid sample of 339 (see Table 2).

**Table 2:** Demographic profile of the respondents (*N* = 339).

Variables	Category	Frequency	Percentage
Gender	Male	222	65.5%
	Female	117	34.5%
Age	Under 24	32	9.4%
	25–34	164	48.4%
	35–44	83	24.5%
	45–54	49	14.5%
	Over 55	11	3.2%
Education	High school /Technical school	26	7.7%
	Junior college	72	21.2%
	Undergraduate college	199	58.7%
	Master’s degree or higher	42	12.4%
Occupation	Scientific research / Education / Medical staff	40	11.8%
	Staff of governmental agencies or government-sponsored institutions	67	19.8%
	Company employee	72	21.2%
	Freelance	78	23.0%
	Student	46	13.6%
	Retired personnel	11	3.2%
	Not specified	25	7.4%
Average monthly income	Below 5,000 yuan	56	16.5%
	5,001–10,000 yuan	158	46.6%
	10,001–15,000 yuan	68	20.1%
	15,001–20,000 yuan	26	7.7%
	More than 20,000 yuan	31	9.1%
Average duration of ski tourism per year	Within 10 days	77	22.7%
	11–20 days	131	38.6%
	21–30 days	75	22.1%
	More than 30 days	56	16.6%

The questionnaire data were analyzed *via* SPSS 18.0. The Cronbach’s alpha of the total number of questionnaires is 0.987. The Cronbach’s alpha of the destination attribute subscale is 0.960. The Cronbach’s alpha of the experience result subscale is 0.957. The Cronbach’s alpha of the personal value subscale is 0.954. The reliability of the questionnaire is considered good. Exploratory factor analysis was first performed to identify the dimensions of latent variables, followed by OLS-based regression analysis to investigate the relationship among the different dimensions.

## Results

### Exploratory factor analysis

Kaiser–Meyer–Olkin test and Bartlett’s Test of Sphericity were conducted on the survey data’s subscale. The results show that the KMO value was greater than 0.9, while the Bartlett’s Test of Sphericity results show that the significance probability was 0.000 < 0.05. Therefore, the three subscales were suitable for factor analysis. In this study, the principal component analysis method was used to do factor analysis on the three subscales: destination attribute, experience consequence, and personal value. The common factors with feature root greater than 1 were extracted by the varimax rotation. The items with a factor load coefficient less than 0.5 were deleted. Meanwhile, if two or more factor loads of a certain item are greater than 0.5, it is considered that there may be cross-loading, and the item will also be deleted (Lai, 2004).

#### Factor analysis of ski tourism destination attributes

Five common factors were extracted from the ski tourism destination attribute subscale, with the cumulative interpretation degree of the total variance at 77.520%. The factor loading of the item traffic accessibility in the original scale is less than 0.5. Therefore, it is deleted. A total of 19 items were retained. The five common factors extracted are as follows (see Table 3). And it should be noted that values of the factor-level variables were obtained *via* computing the average of their corresponding items.

**Table 3:** Factor loading of destination attributes.

Destination attributes	Factor loading	Mean	SD	Cronbach’s alpha
**Ski resort conditions**		3.93	0.95	0.93
Length of ski track	0.81	3.91	1.09	
Slope of snow track	0.76	3.83	1.06	
Snow quality and snow condition	0.75	4.02	1.27	
Width of ski track	0.73	3.79	1.21	
Crowding degree of ski resort	0.65	3.95	1.15	
Scale of ski resort	0.58	3.92	1.14	
Ski infrastructure	0.53	4.12	1.09	
**Leisure and entertainment**		3.32	1.12	0.83
Tourism shopping	0.88	3.02	1.37	
Leisure and entertainment facilities	0.85	3.32	1.32	
Ski resort popularity	0.59	3.62	1.20	
**Price and cost**		3.75	1.06	0.91
Ticket price of ski resort	0.75	3.97	1.17	
Transportation cost	0.71	3.72	1.22	
Accommodation cost	0.71	3.69	1.15	
Catering cost	0.71	3.64	1.23	
**Services and supporting facilities**		3.74	1.07	0.86
Catering facilities	0.70	3.58	1.16	
Ski resort service	0.58	4.12	1.09	
Accommodation facilities	0.57	3.78	1.30	
**Destination scenery**		3.54	1.13	0.81
Destination natural scenery	0.75	3.61	1.14	
Destination cultural scenery	0.61	3.46	1.33

1. Ski resort conditions. This factor describes the environment and facilities of the ski resort and includes seven items: the length of ski track, the width of ski track, the slope of the snow track, snow quality and snow condition, crowding degree of the ski resort, the scale of ski resort and ski infrastructure.
2. Leisure and entertainment. This factor describes leisure and entertainment facilities and includes three items: tourism shopping, leisure and entertainment facilities and ski resort popularity.
3. Price and cost. This factor describes incurred expenses and includes four items: ticket price of the ski resort, accommodation cost, transportation cost, and catering cost.
4. Services and supporting facilities. This factor describes ski tourism support amenities and service management and includes three items: catering facilities, accommodation facilities, and ski resort service.
5. Destination scenery. This factor describes the natural and cultural scenery of the ski tourism destination and includes two items: the destination’s natural scenery and the destination’s cultural scenery.

#### Factor analysis of experience consequences of ski tourism enthusiasts

Three common factors were extracted from the subscale of ski tourism enthusiasts’ experience consequence, with the cumulative interpretation degree of the total variance at 79.68%. The factor load of high flexibility in the original scale was less than 0.5, so it was deleted. There was cross-loading in the six items of trust, worry-free stimulation, high playability, high safety, and novelty, so it was also deleted. Eight items were retained at last. Three common factors extracted were as follows (see Table 4).

**Table 4:** Factor loading of experience consequences.

Experience consequences	Factor loading	Mean	SD	Cronbach’s alpha
**Embodied experience**		3.82	0.94	0.84
Relaxation	0.81	3.76	1.09	
Close to nature	0.79	3.86	1.10	
High comfort level	0.68	3.84	1.05	
**Value experience**		3.84	1.03	0.85
Improving skills	0.85	3.71	1.07	
Saving time	0.79	3.82	1.21	
High-cost performance	0.65	4.01	1.24	
**Interpersonal relationship experience**		3.45	1.17	0.82
Getting attention	0.91	3.41	1.32	
Social atmosphere	0.70	3.48	1.22

1. Embodied experience. This factor describes the direct experiences of ski tourists and includes three items: relaxation, close to nature and high comfort level.
2. Value experience. This factor describes time, skills and other gains perceived by ski tourism enthusiasts and includes three items: improving skills, saving time and high-cost performance.
3. Interpersonal relationship experience. This factor describes social and interpersonal experiences gained by ski tourism enthusiasts and includes two items: getting attention and social atmosphere.

#### Factor analysis of the personal value of ski tourism enthusiasts

Three common factors were extracted from the personal value subscale of ski tourism enthusiasts, with the cumulative interpretation degree of the total variance at 79.956%. The three items of self-challenge, sense of belonging and sense of freedom in the original scale had cross load, so they were deleted. Nine items were retained at last. See Table 5 for the three common factors extracted.

**Table 5:** Factor loading of personal values.

Personal value	Factor loading	Mean	SD	Cronbach’s alpha
**Positive emotion**		3.84	0.97	0.91
Sense of security	0.79	3.94	1.22	
Happiness and pleasure	0.78	3.80	1.10	
Joy of life	0.74	3.80	1.19	
Sense of satisfaction	0.70	3.95	1.07	
Sense of achievement	0.60	3.73	1.07	
**Self-development**		3.70	1.00	0.85
Self-realization	0.82	3.69	1.09	
Self-improvement	0.75	3.71	1.07	
**Self-adjustment**		3.62	1.10	0.81
Promoting work and learning	0.90	3.59	1.29	
Spiritual comfort	0.65	3.65	1.11

1. Positive emotion. This factor describes the positive emotional experiences of ski tourists and includes five items: a sense of security, happiness and pleasure, the joy of life, a sense of satisfaction, and a sense of achievement.
2. Self-development. This factor describes the personal growth of ski tourism enthusiasts and includes two items of self-realization and self-improvement.
3. Self-adjustment. This factor describes the role of ski tourism in eliminating negative effects of life and promoting the development of work, study, and mental health and includes two items: promoting work and learning and spiritual comfort.

### Linear regression model

In order to verify the correlation between the factors of the three subscales (destination attribute, personal experience consequence, and personal value) and further explore how the destination attributes affect the ski experience consequences and personal values, this study uses the linear regression model to conduct regression analysis on each hypothetical path. The values of variance of inflation factor (VIF) and tolerance for each variable all indicate that there is no multi-collinearity within the independent variables (VIF values <10, the values of tolerance >0.1).

#### Linear regression model of the impact of ski tourism destination attributes on experience consequences

To study the impact of ski tourism destination attributes on experience consequences, this study took ski resort conditions, leisure and entertainment, price and cost, services and supporting facilities, and destination scenery at the level of ski destination attributes as independent variables while embodied experience, value experience and interpersonal relationship experience at the level of experience consequences are considered as dependent variables for linear regression model (see Table 6).

**Table 6:** Linear regression model.

Predictors	Dependent variables		
	Embodied experience	Value experience	Interpersonal relationship experience
Norm	**(4.75)**	**(1.17)**	**(−1.41)**
Ski resort conditions	**0.39** ^**^ **(7.37)**	**0.53** ^**^ **(10.21)**	
Leisure and entertainment	**0.09** ^*^ **(2.25)**		**0.39** ^**^ **(8.44)**
Price and cost	**0.16** ^**^ **(3.41)**	**0.34** ^**^ **(7.25)**	**0.32** ^**^ **(5.89)**
Services and supporting facilities	**0.26** ^**^ **(4.73)**		
Destination scenery	**0.21** ^**^ **(4.78)**	**0.09** ^*^ **(0.09)**	
Model statistics	*R*^2^ = 0.75 Adj. *R*^2^ = 0.75\F = 199.29 *N* = 339	*R*^2^ = 0.76 Adj. *R*^2^ = 0.75\F = 208.39 *N* = 339	*R*^2^ = 0.67 Adj. *R*^2^ = 0.67\F = 136.62 *N* = 339

The values appearing in bold are considered significant (*p < 0.05, **p < 0.001). Numbers in parenthesis are *t* values.

1. The influence of ski tourism destination attribute on embodied experience.

As shown in Table 6, price and cost, leisure and entertainment, service and support facilities, and destination scenery all have a significant positive impact on embodied experience. By comparing the coefficient of the regression equation, it can be seen that the influence of ski resort conditions on embodied experience is the largest (*β* = 0.39), followed by service and supporting facilities (*β* = 0.26). And the influence of leisure and entertainment on embodied experience is the smallest (*β* = 0.09).

2. The influence of ski tourism destination attributes on value experience.

According to the coefficient test results of the regression equation, ski resort conditions, price and cost, and destination scenery have a significant positive impact on the value experience. Among them, ski resort conditions have the greatest impact on value experience (*β* = 0.53), followed by price and cost (*β* = 0.34). Destination scenery has the least impact (*β* = 0.09). Leisure and entertainment services and supporting facilities have an insignificant effect on the value experience.

3. The influence of ski tourism destination attributes on interpersonal relationship experience.

The results show that leisure and entertainment and price and cost have a significant positive impact on the interpersonal relationship experience. Among them, leisure and entertainment (*β* = 0.39) have a greater impact on interpersonal relationship experience than price and cost (*β* = 0.32). The regression coefficients of ski resort conditions, services and supporting facilities, and destination scenery are significantly greater than 0.05, which has no significant impact on the interpersonal relationship experience.

#### Linear regression model of the impact of ski tourism enthusiasts’ experience consequences on personal values

To study the influence of tourism experience consequences on personal values, this study took embodied experience, value experience, and interpersonal relationship experience as independent variables and took positive emotion (self-development and self-adjustment) as dependent variables for linear regression model (see Table 7).

**Table 7:** Linear regression analysis.

Predictors	Dependent variables		
	Positive emotion	Self-development	Self-adjustment
Norm	**(2.41)**	**(2.96)**	**(1.01)**
Embodied experience	**0.49** ^**^ **(12.19)**	**0.27** ^**^ **(5.09)**	**0.29** ^**^ **(5.59)**
Value experience	**0.37** ^**^ **(8.94)**	**0.47** ^**^ **(8.60)**	**0.18** ^**^ **(3.36)**
Interpersonal relationship experience	**0.19** ^**^ **(3.42)**	**0.14** ^**^ **(3.04)**	**0.45** ^**^ **(10.13)**
Model statistics	*R*^2^ = 0.80 Adj.*R*^2^ = 0.80\F = 451.66 *N* = 339	*R*^2^ = 0.65 Adj.*R*^2^ = 0.66\F = 207.79 *N* = 339	*R*^2^ = 0.67 Adj.*R*^2^ = 0.67\F = 230.87 *N* = 339

The values appearing in bold are significant (*p < 0.05, **p < 0.001). Numbers in parenthesis are *t* values.

1. The influence of experience consequences on positive emotion.

The results showed that embodied experience, value experience, and interpersonal relationship experience have a significant positive impact on positive emotion. Among them, embodied experience has the greatest impact on positive emotion (β = 0.49), followed by value experience (*β* = 0.37), and interpersonal relationship experience has the least impact (*β* = 0.19).

2. The influence of experience consequences on self-development.

It can be seen from Table 7 that embodied experience, value experience, and interpersonal relationship experience have a significant positive impact on self-development. Among them, value experience has the greatest impact on self-development (*β* = 0.47), followed by embodied experience (*β* = 0.27), while interpersonal relationship experience has the least impact (*β* = 0.14).

3. The influence of experience consequence on self-adjustment.

As can be seen from Table 7, embodied experience, value experience, and interpersonal relationship experience have a significant positive impact on self- adjustment. Among them, interpersonal relationship experience has the greatest impact on self-adjustment (*β* = 0.45), followed by embodied experience (*β* = 0.29), and value experience has the least impact (*β* = 0.18).

### Value hierarchy diagram of influencing factors of ski tourism enthusiasts’ destination choice

**Figure 1:**
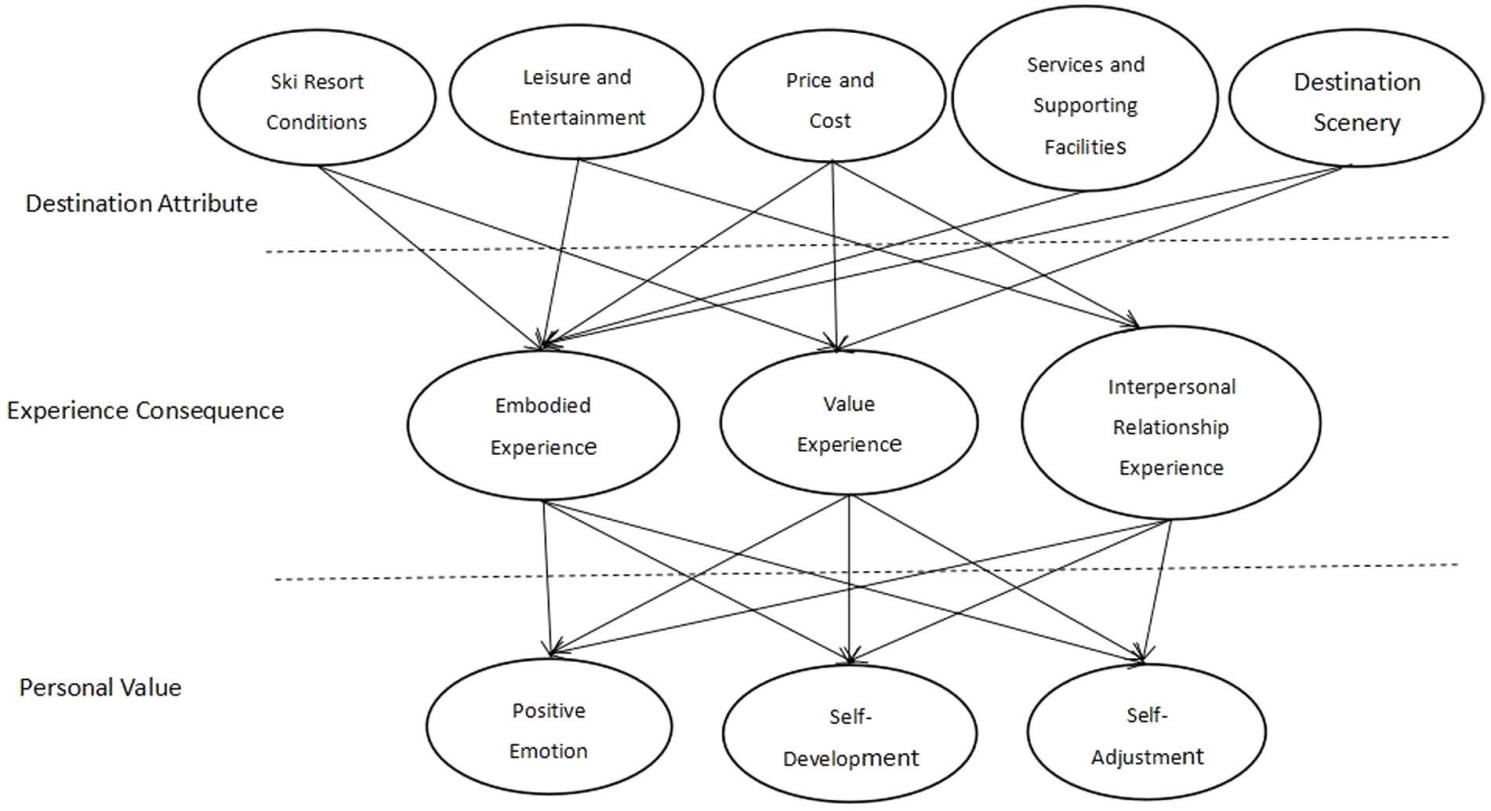
Value hierarchy diagram of influencing factors of ski tourism enthusiasts’ destination choice.

Through qualitative analysis, this study firstly obtained the influencing factors of ski tourism enthusiasts’ destination choice, including destination attributes, experience consequences, and personal values and followed up by further investigation using a survey. Through factor analysis of the survey data and linear regression model of the extracted main factors, the internal correlation among the three levels of influencing factors was obtained. See Table 1 for the value hierarchy diagram of influencing factors of skiing tourism enthusiasts’ destination choice (Figure 1).

## Discussion

Various factors at the level of ski tourism destination attributes have an impact on the experience consequence of ski tourism enthusiasts. Among them, the ski resort condition is the primary factor affecting the embodied experience and value experience at the experience consequence level. It is also noted that for ski tourism enthusiasts, the importance of interpersonal relationship experience is less than embodied experience and value experience. Overall, at the level of ski tourism destination attribute, ski resort condition is the most valued attribute of ski tourism enthusiasts with the strongest impact on their experience consequences.

According to the results of the factor analysis, ski resort conditions include seven specific items: snow quality and snow condition, the length of ski track, the slope of snow track, the width of ski track, crowding degree of ski resort, the scale of ski resort and ski infrastructure. Since the main purpose of ski tourism enthusiasts is to ski, and their degree of specialization is higher than that of mass tourists, their requirements for ski resort conditions are also correspondingly higher (Won et al., 2008). When the ski resort facilities and service level can meet the needs of skiing tourism enthusiasts, they can feel the relaxation brought about by skiing comfort and other personal experiences. Previous studies have shown that the number, slope and challenge of ski trails are the primary factors for ski tourists to consider when choosing a destination (Miragaia and Martins, 2015). The higher the professionalism of ski tourists, the more stringent the requirements for ski resort conditions such as ski trails (Richards, 1996; Godfrey, 1999; Dickson and Faulks, 2007).

While leisure and entertainment at the level of destination attribute is the strongest predictor of interpersonal relationship experience at the experience result level, both are the least valued factors by ski tourism enthusiasts. This, to some extent, corresponds to Konu et al.’s (2011) observation that compared with active ski tourists, passive participants in ski tourism, such as older family-oriented tourists, would pay more attention to the experience result of interpersonal relationship experience. As active ski tourists, enthusiasts focus their attention on aspects relating to skiing *per se*, while other aspects such as entertainment and social needs are secondary (Miragaia and Martins, 2015).

The analysis results also show that the embodied experience, value experience, and interpersonal relationship experience at the experience result level all significantly impact on the positive emotion, self-development, and self-adjustment at the personal value level. Previous studies mentioned that relieving stress, a sense of achievement, and entertainment and leisure are tourists’ main motivations for choosing ski tourism (Klenosky et al., 1993; Liu and Dong, 2013). Apart from these motivations (as represented by the value needs of positive emotion and self-adjustment), ski enthusiasts also show a strong need for self-development. And value experience is identified as the strongest predictor of their self-development. Specifically, through skill improvement and perceived value, ski tourists obtain a sense of self-transcendence, self-improvement, and self-realization, meeting the need for self-development.

## Conclusion

Based on the means-end chain theory, this study used the mixed-method approach to explore the factors affecting the destination choice of ski tourism enthusiasts. First of all, this study adopts ski tourism enthusiasts as the research object, thereby broadening the research horizon of ski tourism and tourists. Secondly, this study attempts to explore the value link mode of destination choice behavior of ski tourism enthusiasts by combining soft laddering interviews and quantitative questionnaire, further revealing how the objective attributes of the ski tourism destination affect the value feelings of ski tourism enthusiasts on experience consequences, expands the application scope of means-end chain theory, and enriches the theoretical system of ski industry development. Thirdly, according to the characteristics of destination choice behavior of ski tourism enthusiasts, this study puts forward marketing suggestions for ski tourism destinations, which are of great significance to guide the design and marketing of ski tourism destinations, improve consumer loyalty to destinations, and promote the sustainable development of the ski industry.

This study is not without limitations. Given the exploratory nature of this study, exploratory factor analysis was adopted to probe into the dimensions of different levels of factors concerning the destination choice of ski tourism enthusiasts, followed by regression analysis to examine the relationship among the different levels of factors. Future studies may employ more rigorous methods such as the structural equation modeling approach to examine our findings. Moreover, the non-probability sampling approaches adopted in this study, be it the snowball sampling to approach interviewees or the convenience sampling to obtain questionnaire survey respondents, may undermine the representativeness of our sample and the applicability of our findings. Future research may examine the current results with probability sampling approaches and with larger samples.

## Data availability statement

The original contributions presented in the study are included in the article/supplementary material, further inquiries can be directed to the corresponding author.

## Author contributions

Conceptualization, HS and XX; data curation, HS and LZ; formal analysis, HS and XX; funding acquisition, HS and XX; investigation, LZ; methodology, JG; original draft, HS and XX; review and editing, JG. All authors have read and agreed to the published.

## Funding

This work was supported by the Key Laboratory of the Sustainable Development of Xinjiang’s Historical and Cultural Tourism, grant number: 2020D04105; Key Projects of Philosophy and Social Sciences Research in Xinjiang University, grant number: 22APY016; and the National Natural Science Foundation of China, grant number: 42261041.

## Conflict of interest

The authors declare that the research was conducted in the absence of any commercial or financial relationships that could be construed as a potential conflict of interest.

## Publisher’s note

All claims expressed in this article are solely those of the authors and do not necessarily represent those of their affiliated organizations, or those of the publisher, the editors and the reviewers. Any product that may be evaluated in this article, or claim that may be made by its manufacturer, is not guaranteed or endorsed by the publisher.
